# Optimum porphyrin accumulation in epithelial skin tumours and psoriatic lesions after topical application of **δ**-aminolaevulinic acid

**DOI:** 10.1038/sj.bjc.6690255

**Published:** 1999-03

**Authors:** C Fritsch, P Lehmann, W Stahl, K W Schulte, E Blohm, K Lang, H Sies, T Ruzicka

**Affiliations:** Institute of Physiological Chemistry, Heinrich-Heine-University Düsseldorf, Düsseldorf, Germany; Department of Dermatology, Heinrich-Heine-University Düsseldorf, Düsseldorf, Germany

**Keywords:** aminolaevulinic acid, basal cell carcinoma, squamous cell carcinoma, psoriasis, photodynamic therapy, porphyrin metabolites

## Abstract

Photodynamic therapy with topically applied δ-aminolaevulinic acid is used to treat skin tumours by employing endogenously formed porphyrins as photosensitizers. This study examines the time course of porphyrin metabolite formation after topical application of δ-aminolaevulinic acid. Porphyrin biosynthesis in human skin tumours (basal cell carcinoma, squamous cell carcinoma), in psoriatic lesions, and in normal skin was investigated. Skin areas were treated with δ-aminolaevulinic acid, and levels of total porphyrins, porphyrin metabolites and proteins were measured in samples excised after 1, 2, 4, 6, 9, 12 and 24 h. There was an increase in porphyrin biosynthesis in all tissues with maximum porphyrin levels in tumours between 2 and 6 h and in psoriatic lesions 6 h after treatment. The pattern of porphyrins showed no significant difference between normal and neoplastic skin, protoporphyrin being the predominant metabolite. The results suggest that optimum irradiation time for superficial epithelial skin tumours may be as soon as 2 h after application of δ-aminolaevulinic acid, whereas for treatment of psoriatic lesions an application time of 6 h is more suitable. © 1999 Cancer Research Campaign


*British Journal of Cancer* (1999) **79**(9/10), 1603–1608
© 1999 Cancer Research Campaign
Article no. bjoc.1998.0255

### Optimum porphyrin accumulation in epithelial skin tumours and psoriatic lesions after topical application of δ-aminolaevulinic acid

**C Fritsch[1,2], P Lehmann[2], W Stahl[1], KW Schulte[2], E Blohm[2], K Lang[2], H Sies[1] and T Ruzicka[2]**

[1]Institute of Physiological Chemistry I; [2]Department of Dermatology, Heinrich-Heine-University Düsseldorf, Düsseldorf, Germany

**Summary** Photodynamic therapy with topically applied δ-aminolaevulinic acid is used to treat skin tumours by employing endogenously formed porphyrins as photosensitizers. This study examines the time course of porphyrin metabolite formation after topical application of δ-aminolaevulinic acid. Porphyrin biosynthesis in human skin tumours (basal cell carcinoma, squamous cell carcinoma), in psoriatic lesions, and in normal skin was investigated. Skin areas were treated with δ-aminolaevulinic acid, and levels of total porphyrins, porphyrin metabolites and proteins were measured in samples excised after 1, 2, 4, 6, 9, 12 and 24 h. There was an increase in porphyrin biosynthesis [illegible] in all tissues with maximum porphyrin levels in tumours between 2 and 6 h and in psoriatic lesions 6 h after treatment. The pattern of porphyrin [illegible]s showed no significant difference between normal and neoplastic skin, protoporphyrin being the predominant metabolite. The results suggest that optimum irradiation time for superficial epithelial skin tumours may be as soon as 2 h after application of δ-aminolaevulinic acid, whereas for treatment of psoriatic lesions an application time of 6 h is more suitable.

**Keywords:** aminolaevulinic acid; basal cell carcinoma; squamous cell carcinoma; psoriasis; photodynamic therapy; porphyrin metabolites

Photodynamic therapy (PDT) with δ-aminolaevulinic acid (ALA) is based on the administration of ALA, the non-fluorescent first committed compound in the pathway of haem biosynthesis, to the diseased skin (Kennedy et al, 1990). Thus, ALA-synthase is bypassed and porphyrins as photosensitizers are formed, preferentially in neoplastic tissues. Subsequent light irradiation of the sensitized tissue leads to reactive species-induced tumour necrosis by targeting biological membranes (Pass, 1993) and by vascular damage with subsequent tumour cell anoxia (Goetz et al, 1990).

In dermatology, topical ALA-PDT has been used to treat precancerous lesions and neoplasms of the skin without serious adverse effects (Fritsch et al, 1996*b*). High efficacy of ALA-PDT was achieved for solar keratoses, superficial basal cell carcinomas, and superficial squamous cell carcinomas (Kennedy et al, 1990; Wolf et al, 1993; Calzavara-Pinton, 1995; Fritsch et al, 1996*a*, 1997*d*). In addition, there are also reports that psoriatic plaques can be beneficially treated with ALA-PDT (Boehncke et al, 1994; Stringer et al, 1996).

The presence of high intralesional porphyrin levels is important for effective ALA-PDT. The amount of newly formed porphyrins depends on the application time of ALA. Distribution studies using porphyrin fluorescence revealed maximum fluorescence in tumours up to 24 h after topical or systemical administration of ALA (Kennedy et al, 1990; Abels et al, 1994; Szeimies et al, 1994; Martin et al, 1995; Peng et al, 1995; Kriegmair et al, 1996). However, biochemical data on the absolute levels of porphyrin metabolites, which have been determined in untreated skin (Goerz et al, 1995), are lacking for ALA-treated lesions. In addition, the time-point of maximum porphyrin accumulation is still not exactly known for specific cutaneous lesions. Therefore, we examined the time-dependent formation of porphyrins after topical application of ALA in epithelial skin tumours, psoriatic lesions and normal skin.

### MATERIALS

Tissue samples used were: basal cell carcinomas (BCC; $n$ = 32), squamous cell carcinomas (SCC; $n$ = 32), psoriatic lesions (PS; $n$ = 32), normal skin (NS; $n$ = 160). Tumour samples were obtained from patients who underwent surgery. Psoriatic tissue samples were taken by punch biopsy for histopathological examination and analysis of the porphyrin pattern.

Normal skin was examined: (a) from patients with tumours or psoriasis which were treated with ALA and (b) from subjects free of tumour or psoriasis. Tissue samples of normal skin from patients with tumours or psoriasis were either taken from the tumour- or psoriasis-adjacent site (NS-A) or from locations distant from the lesions (NS-D) = individual controls. Additionally, normal skin samples (NS) were obtained from patients free of BCC, SCC or PS = interindividual controls (Table 1). This study design was chosen to investigate the influence of the proximity of normal skin to neoplastic or psoriatic skin on ALA-induced porphyrin accumulation. Each patient received comprehensive information about the scope of the study.

### METHODS

### ALA-treatment and photodynamic diagnosis (PDD)

Pilot studies on topical ALA application (up to 80 mg $cm^{-2}$) to normal skin revealed plateau porphyrin levels (approximately 4 nmol $g^{-1}$ protein) > 40 mg ALA $cm^{-2}$ (data not shown). Based on

*Received 3 December 1997*
*Revised 21 September 1998*
*Accepted 20 October 1998*

*Correspondence to:* C Fritsch

**Table 1** Design of the study: selection of normal skin samples

| Disease | Sample from the same patient<br>Lesion<br>*n* | Adjacent to lesion<br>NS-A<br>*n* | Distant from lesion<br>NS-D<br>*n* | Samples from other patients<br>No lesion<br>NS<br>*n* |
|---|---|---|---|---|
| BCC | 32 | 16 | 16 | |
| SCC | 32 | 16 | 16 | 32 |
| Psoriasis | 32 | 32 | 32 | |

BCC, basal cell carcinoma; SCC, squamous cell carcinoma; PS, psoriasis lesion; NS-A, normal skin adjacent to lesion; NS-D, normal skin distant from lesion; NS, normal skin in patients free of lesions. Normal skin samples were taken from patients whose tumours or psoriatic lesions were also treated by ALA. One part of these samples was adjacent to the lesions (NS-A) and one part distant to the lesions (NS-D). In addition, normal skin samples were examined in other patients which were not yet included for tumours or psoriasis (NS). Thus, the neoplastic lesions had individual and interindividual controls of normal skin.

**Table 2** Fluorescence intensities of tissues treated by 20% ALA

| | 1 h | 2 h | 4 h | 6 h | 9 h | 12 h | 24 h |
|---|---|---|---|---|---|---|---|
| NS | 0.8 ± 0.2 | 0.8 ± 0.2 | 0.8 ± 0.2 | 1.3 ± 0.2 | 1.5 ± 0.3 | 1.5 ± 0.3 | 1.5 ± 0.3 |
| BCC | 2.8 ± 0.2 | 2.6 ± 0.2 | 2.8 ± 0.2 | 2.9 ± 0.1 | 2.6 ± 0.2 | 2.8 ± 0.2 | 2.5 ± 0.2 |
| SCC | 2.8 ± 0.2 | 2.5 ± 0,3 | 3.0 ± 0 | 2.8 ± 0.2 | 2.5 ± 0.3 | 2.5 ± 0.3 | 2.3 ± 0.2 |
| Psoriasis | 1.3 ± 0.2 | 1.8 ± 0.4 | 2.8 ± 0.2 | 2.8 ± 0.2 | 2.5 ± 0.3 | 2.5 ± 0.3 | 1.5 ± 0.3 |

Fluorescence intensities of ALA-treated skin irradiated by Wood's light (370–405 nm, 10-cm distance, 5 mW $cm^{-2}$). Values are given according to a fluorescence standard: 0 = no fluorescence, 3 = maximum fluorescence ($n$ = 4; mean ± s.e.m.). Normal skin (NS) was observed in patients free of tumours and psoriasis. NS, BCC, SCC or PS not treated by ALA did not show any fluorescence.

these data and due to clinical experience in ALA-PDT (Calzavara-Pinton, 1995; Wolf et al, 1993) an ALA (hydrochloride) mixture of 20% was prepared in an ointment (Neribas®, Schering, Berlin, Germany). Of the 20% ALA mixture, 0.2 g (40 mg ALA $cm^{-2}$) were applied to a 1-$cm^2$ skin area of BCC, SCC, psoriatic lesions, and all normal skin lesions. Treated skin was covered with an occlusive foil (Tegaderm®, 3M Healthcare, Borken, Germany), gauze, aluminium foil and tape to enhance tissue penetration and avoid photobleaching of the formed porphyrins. After defined incubation times (1, 2, 4, 6, 9, 12 and 24 h) the tape and the ointment were removed and the treated area was illuminated with Wood's light (Fluotest®, Xenotest, Hanau, Germany; 370–405 nm). Fluorescence intensity was measured and expressed semiquantitatively according to a fluorescence standard as 0 (no), 1 (low), 2 (medium) and 3 (strong fluorescence). The fluorescent area was marked. Basal values were obtained from untreated controls.

According to our clinical experience, BCC and SCC reveal a strong, homogeneous, ALA-induced porphyrin fluorescence under Wood's light (Figure 1A) (Fritsch et al, 1996*a*, 1997*b*), whereas about 20–40% of psoriatic lesions show areas with inhomogeneous or absent fluorescence (Figure 1B). For better comparison of intralesional porphyrin formation in PS and tumours, we selected only uniform fluorescing psoriatic areas for our study. Biochemical analysis was also performed in nonfluorescing psoriatic areas ($n$ = 8) which were treated with ALA for 6 h.

### Preparation of skin samples

Only superficial layers of skin samples (< 1 mm) were included in the study due to the limited penetration of topically applied ALA (Szeimies et al, 1994; Martin et al, 1995; Peng et al, 1995). Immediately after excision tissue samples were frozen in liquid nitrogen and stored at – 80°C until further examination.

### Determination of total porphyrin and protein levels in tissues

Tissue samples were weighed and cut into small pieces. After homogenization with an Ultraturrax and centrifugation at 3000 U $min^{-1}$ for 10 min, porphyrins were isolated with 1.0 N perchloric acid/methanol (1/1, v/v). In the supernatant, the total porphyrin level was assessed by fluorescence spectroscopy (Perkin Elmer LS-5, Überlingen, Germany); emission was recorded in a range of 520–700 nm at an excitation wavelength of 405 nm (Soret band). For quantification a protoporphyrin standard (Porphyrin Products Inc., Logan, Utah, USA) was used (Fritsch et al, 1997*a*). Protein levels were determined in the pellet according to Lowry et al (1951).

### Determination of porphyrin metabolites in tissues

The supernatant was adjusted with acetic acid to pH 3–4, porphyrins were bound to talcum, esterified and metabolites were identified by HPLC with fluorescence detection (L-7480, Merck Hitachi, Darmstadt, Germany) using a porphyrin standard mixture (Porphyrin Products Inc., Logan, Utah, USA) for quantification (Seubert and Seubert, 1982). The following metabolites were analysed: protoporphyrin, tricarboxylic porphyrin, coproporphyrin, pentacarboxylic porphyrin, hexacarboxylic porphyrin, heptacarboxylic porphyrin and uroporphyrin. The last four porphyrin metabolites are designated as highly carboxylated porphyrins; data are given as the sum of these compounds.

 © Cancer Research Campaign 1999

A

B

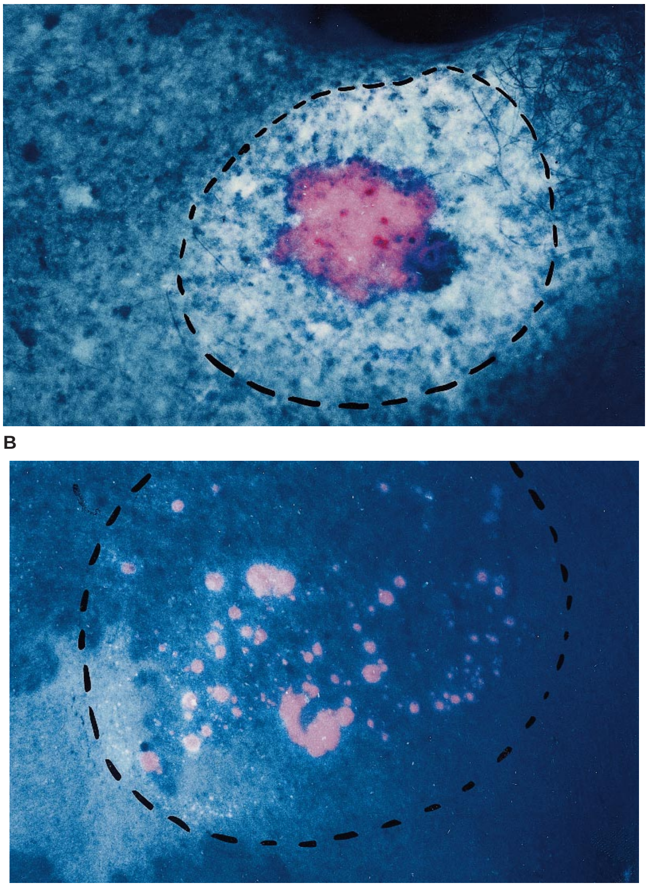

**Figure 1** Lesions treated for 6 h with 20% ALA and illuminated with Wood's light. Black dotted lines indicate the areas treated by ALA. (**A**) BCC showing a homogeneous bright red fluorescence of porphyrins formed intralesionally. Tumour-surrounding tissue treated by ALA also shows fluorescence though less intense, impressing as whitish or pale pink. (**B**) Psoriatic lesion with stippled bright fluorescent areas indicating non-uniform sensitization with porphyrins

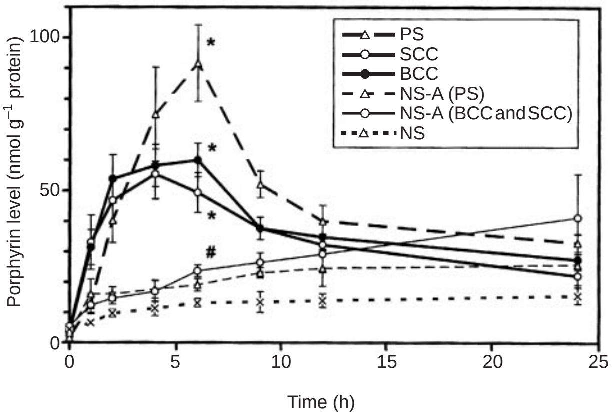

**Figure 2** Porphyrin levels in BCC, SCC, PS, NS adjacent to tumours [NS-A (BCC and SCC)], NS adjacent to PS [NS-A (PS)] and NS of patients not included for ALA treatment of tumour or psoriasis. In all tumour and psoriatic tissues, the maximum porphyrin accumulation was detected at 4–6 h after ALA application (*$P < 0.005$ BCC, SCC, PS vs NS). NS-A showed higher porphyrin levels than NS obtained from patients free of lesions, respectively (†$P < 0.05$). Porphyrin levels in NS distant from lesions (NS-D) were not different from those measured in NS (data are not included in the figure). Mean ± s.e.m. [$n = 4$; except NS-A (BCC, SCC): $n = 8$ for each data point]

### Statistical calculation

Statistical analysis of the data was performed by Student's *t*-test. Data are reported as mean ± SEM. Changes were considered statistically significant when $P < 0.05$.

### RESULTS

### Fluorescence intensities of treated tissues

The highest macroscopic fluorescence intensities were found in BCC, SCC and PS 4–6 h after ALA application. At all time-points normal skin (NS) revealed only slight fluorescence intensities compared with neoplastic and psoriatic skin (Table 2).

### Basal total porphyrin levels in BCC, SCC, PS and skin (all)

In untreated tissues (basal values), the levels of total porphyrins were similarly low in normal skin, tumours and psoriasis (Figure 2).

### Total porphyrin levels in BCC, SCC, PS and skin after topical application of ALA

ALA application induced porphyrin synthesis in all tissues. The porphyrin levels in BCC and SCC showed maximum values between 2 h (53.8 ± 19.3 and 46.8 ± 17.3) and 6 h (60.0 ± 13.4 and 49.4 ± 13.1 nmol $mg^{-1}$ protein). The highest porphyrin accumulation was measured in psoriatic lesions with 91.7 ± 14.4 nmol $mg^{-1}$ protein at 6 h. Normal skin samples: in NS and NS-D, the accumulation of ALA-induced porphyrins was comparably low with a maximum at 24 h (15.6 ± 6.6 nmol $mg^{-1}$ protein) (Figure 2); at 6 h, NS-A close to tumours or PS showed higher porphyrin levels (23.8 ± 4.0 and 19.1 ± 4.1 nmol $mg^{-1}$ protein) than NS or normal skin distant from lesions (NS-D). The ratio of porphyrins measured in BCC vs NS (BCC/NS) showed maximum values between 1 and 6 h (4.8–5.5) (Figure 3). The maximum ratio of porphyrins SCC/NS (4.8–5.1) was observed 1–4 h after ALA-application. In PS, there was the most distinct maximum of the porphyrin ratio to NS with 7.0 after 6 h. However, using normal skin adjacent to tumours of PS (NS-A) as reference, the ratios of porphyrin levels in lesions vs skin were much lower; maximum values were measured after 2 h for BCC (3.7), after 4 h for SCC (3.3) and after 6 h for PS (4.8).

### Basal porphyrin metabolite levels in BCC, SCC, PS and NS (all)

The pattern of porphyrin metabolites was comparable in all untreated tissues with protoporphyrin as the predominant metabolite (82.9–92.3%) followed by uroporphyrin (all tissues) (4.6–6.2%), heptacarboxylic porphyrin (NS: 0.9% and PS: 1.5%), and coproporphyrin (BCC: 2.7% and SCC: 3.9%).

### Porphyrin metabolite patterns in BCC, SCC, PS and NS (all) after topical application of ALA

The pattern of metabolites was not changed following ALA-treatment. Protoporphyrin was still the predominant metabolite and

**Table 3** Total porphyrin levels and distribution of porphyrin metabolites 6 h after topical application of 20% ALA

| | Total porphyrins (nmol g–1 protein) | Proto-porphyrin (%) | Tricarboxylic porphyrin (%) | Copro-porphyrin (%) | Pentacarboxylic porphyrin (%) | Hexacarboxylic porphyrin (%) | Heptacarboxylic porphyrin (%) | Uro-porphyrin (%) |
|---|---|---|---|---|---|---|---|---|
| NS | 4.7 ± 0.8 | 88.3 ± 2.8 | 0 ± 0 | 0.6 ± 0.5 | 0 ± 0 | 0.7 ± 0.4 | 4.0 ± 0.9 | 7.9 ± 1.3 |
| BCC | 15.7 ± 1.1* | 92.2 ± 1.2 | 0 ± 0 | 2.1 ± 0.6 | 0 ± 0 | 0.6 ± 0.3 | 1.1 ± 0.4 | 5.3 ± 1.3 |
| SCC | 16.7 ± 2.1* | 92.6 ± 1.7 | 0 ± 0 | 1.9 ± 0.7 | 0 ± 0 | 0.4 ± 0.3 | 3.6 ± 1.1 | 7.1 ± 1.6 |
| PS | 21.9 ± 2.7* | 83.8 ± 7.6 | 0 ± 0 | 1.5 ± 1.3 | 0.5 ± 0.4 | 2.1 ± 1.3 | 4.3 ± 2.7 | 8.9 ± 2.5 |

Data are given as nmol porphyrin g–1 protein (*n* = 4; mean ± s.e.m.; *$P < 0.005$). Porphyrin patterns showed no significant differences between tumours, psoriasis and normal skin.

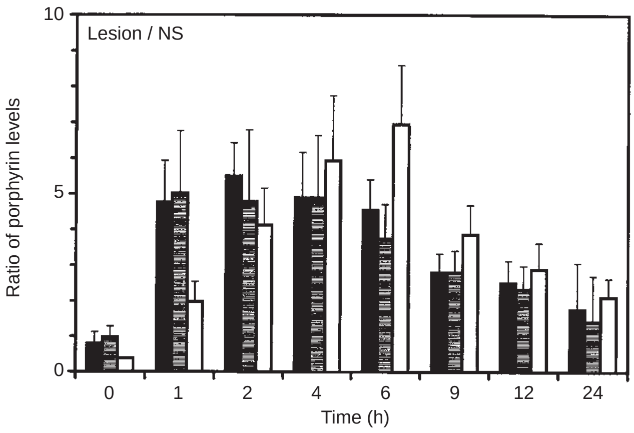

**Figure 3** Ratios of total porphyrin levels BCC/NS (filled bars), SCC/NS (dotted bars), PS/NS (empty bars) after topical application of 20% ALA. Distinct maximum for PS/NS after 6 h. Comparable maximum levels for BCC/NS and SCC/NS between 1 and 4 h

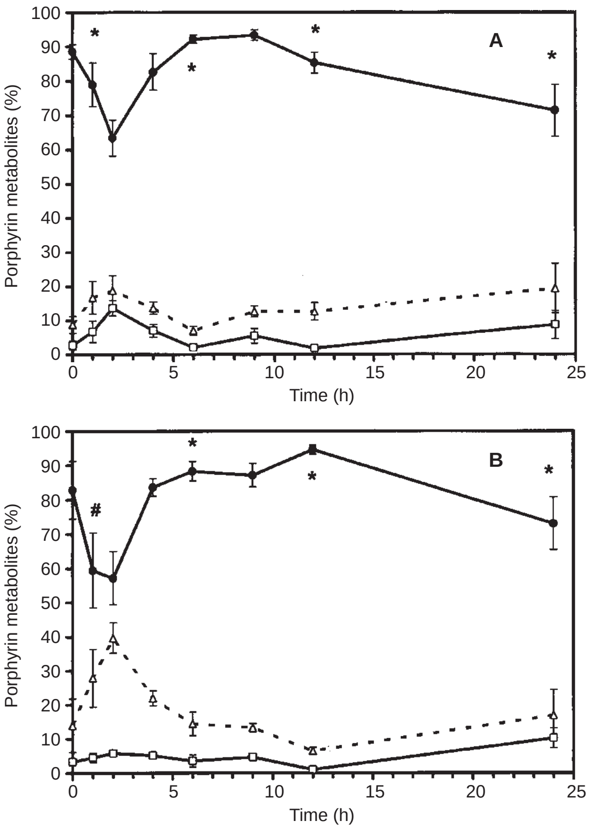

**Figure 4** Distribution of porphyrin metabolites in BCC and NS after topical application of 20% ALA. Protoporphyrin (filled circles), coproporphyrin (open squares), highly carboxylated porphyrins (penta-, hexa-, heptacarboxylic porphyrin, uroporphyrin) (open triangles). (**A**) In BCC, protoporphyrin was the prevailing metabolite (*$P < 0.001$ vs coproporphyrin and the highly carboxylated porphyrin metabolites) (mean ± s.e.m.; *n* = 8 for each data point). (**B**) For NS, a slight decrease of protoporphyrin levels 1 h after ALA application was detected (*$P < 0.001$ and # < 0.01 vs coproporphyrin and the highly carboxylated porphyrin metabolites) (mean ± s.e.m.; *n* = 4 for each data point)

accumulated in all tissues over a period of 24 h (Figure 4A and B). Only at 1 h was there a slight decrease in protoporphyrin and a slight increase in the highly carboxylated porphyrins. At the time-point of the maximum ratio of porphyrins in BCC/NS, SCC/NS or PS/NS (1–12 h, 6 h), protoporphyrin was the prevailing metabolite in the lesions (Table 3, Figure 4A and B).

### DISCUSSION

In the present study the time course of porphyrin metabolite formation in human skin after topical application of ALA was investigated. The maximum levels of porphyrins in epithelial tumours and psoriatic lesions had already been detected at 1–6 h after application of ALA. This indicates that ALA is rapidly absorbed by the damaged skin and abnormal cells, and subsequently converted to porphyrins. In NS and NS-D, porphyrin sensitization was less than in BCC, SCC or PS but prolonged, indicating temporary skin photosensitivity. The higher porphyrin levels in NS-A than in NS (Figure 2) may be due to the leakage of porphyrins from the lesions into the surrounding tissues. This phenomenon may be relevant with respect to PDT efficacy because the tumour-nutrifying tissue is an additional target in therapy (Goetz et al, 1990).

Comparing porphyrin levels in lesions with that in NS (Figures 2 and 3), optimum efficacy of topical ALA-PDT may be expected if irradiation of BCC and SCC is performed between 1 and 6 h, and of PS at 6 h, after ALA treatment.

In general, the available data on ALA-induced porphyrin levels in human tissues are based on fluorescence studies (Warloe et al, 1992; Svanberg et al, 1994; Szeimies et al, 1994; Malik et al, 1995; Martin et al, 1995; Peng et al, 1995; Stringer et al, 1996) (Table 4) and only some biochemical studies were performed (Fritsch et al, 1997*c*, 1998). It was shown by fluorescence microscopy that the major factor limiting the efficacy of topical ALA-PDT is the penetration depth of ALA and ALA-induced

*© Cancer Research Campaign 1999*

**Table 4** Studies on kinetics of ALA-induced porphyrins in various tissues after topical application of ALA

| Human/ animals | Tissue | ALA (%) | Additive | Scale (h) | Max. (h) | Ratio Max. (h) | value | Tissues | Pre-treatment levels (h) | Method | Author |
|---|---|---|---|---|---|---|---|---|---|---|---|
| Human | BCC | 20 | EDTA | 3–48 | 29–48 | n.d. | n.d. | n.d. | n.d. | FM | Peng et al, 1995 |
| | BCC | 20 | – | 4–6 | n.d. | 4–6 | 15 | Tumour/skin | n.d. | LIF | Svanberg et al, 1994 |
| | PS | 20 | – | 1.5–180 | 4–6 | 4–6 | 3–10 | Psoriasis/skin | < 180 | LIF | Stringer et al, 1996 |
| | BCC | 20 | – | 1–24 | 6 | 1–4 | 4.8–5.5 | Tumour/skin | > 24 | SP / HPLC | This study |
| | SCC | 20 | – | 1–24 | 4 | 1–4 | 4.8–5.1 | Tumour/skin | > 24 | SP / HPLC | This study |
| | PS | 20 | – | 1–24 | 6 | 6 | 17 | Psoriasis/skin | > 24 | SP / HPLC | This study |
| Mice | NS | 20 | – | 1–14 | 14 | – | – | – | n.d. | LIF | Peng et al, 1996 |
| | SCC | 20 | – | 2–24 | 10 | 10 | 4.5 | Tumour/skin | 24 | F/FM | Veen et al, 1996 |
| | Mamma Ca* | 20 | – | 1–5 | 3 | 3 | 0.2 | Tumour/skin | n.d. | SP | Peng et al, 1992 |
| | Colon Ca* | 20 | EDTA DMSO | 2–6 | 6 | n.d. | 0.4 | Tumour/skin | n.d. | LIF/SP | Malik et al, 1995 |
| Rat | Bladder Ca | 50 mg | – | 4–24 | 4 | 4 | 2 | Tumour / skin or Tumour/Bladder | 24 | FM | Iinuma et al, 1995 |
| Pig | NS | 20 | – | 0.5–3 | 3 | – | – | – | n.d. | F | Goff et al, 1992 |

*, Subcutaneously growing tumours; Ca, carcinoma; Pre-treatment levels, time-point after ALA-application which was reported to show comparable porphyrin levels prior to ALA treatment; LIF, laser-induced fluorescence analysis; FM, fluorescence microscopy F, fluorescence detection under excitation with UV/visible light; SP, spectrophotometry of extracted porphyrins; HPLC, high performance liquid chromatography; n.d. = no data.

porphyrins, which is dependent on the incubation time of ALA, the combination with additives and the kind of skin surface treated. In the case of BCC, the penetration was limited to a depth of about 0.75–0.81 mm (Martin et al, 1995; Peng et al, 1995) and could be enhanced by addition of dimethylsulphoxide (DMSO) and ethylenediaminetetraacetic acid disodium salt (EDTA) (up to 1.25 mm). Long-time application of ALA together with 4% EDTA for 20–48 h increased the homogeneity of fluorescence distribution, particularly at the bottom of the lesions (Peng et al, 1995).

Fluorescence studies on the kinetics of the ALA-induced porphyrins showed maximum levels in BCC and PS 4–6 h after ALA application with 3–15-fold higher levels than in normal skin (Svanberg et al, 1994; Stringer et al, 1996). These data are supported by our analysis.

In the psoriatic skin samples which showed no fluorescence 6 h after ALA application, we found no higher porphyrin levels (22.4 ± 8.2 nmol $g^{-1}$ protein) than in NS-A. The hyperkeratotic psoriatic areas with limited penetration of ALA may be one reason for the inhomogeneous accumulation of porphyrins, similarly to verrucae vulgares (Fritsch et al, 1997*b*). The correlation of fluorescence intensity under Wood's light with the porphyrin levels suggests that the clinical evaluation of the fluorescence gives an orientation on superficial intralesional porphyrin levels. This method is already used in photodynamic diagnosis (PDD), a novel, effective diagnostic modality in tumour detection and demarcation (Figure 1) (Fritsch et al, 1996*a*, 1997*b*, 1997*d*; Kriegmair et al, 1996).

In the present study no striking differences between basal and ALA-induced porphyrin metabolite patterns were found with protoporphyrin as the predominant compound (Table 3). Inhibition of mitochondrial ferrochelatase, which forms haem by incorporation of iron into protoporphyrin, might decrease haem availability for negative feedback control. This could explain the accumulation of protoporphyrin in tumour and psoriatic tissue, analogous to cells from patients who suffer from erythropoietic protoporphyria (Bloomer et al, 1977). It might be that the normal kinetics of ferrochelatase in epithelial cells, which are most probably responsible for porphyrin synthesis in epithelial neoplasms, are rate-limiting for the entire pathway, and that by supplying an overwhelming quantity of upstream substrate (ALA), protoporphyrin is predominantly accumulated.

The route of ALA administration and the presence of serum proteins may be relevant for increased formation of additional porphyrins such as copro- and uroporphyrin, which was demonstrated in a hamster melanoma (Fritsch et al, 1997*a*), in skin organ cultures (keratoakanthoma, BCC and NS) (Fritsch et al, 1997*c*) and various cell lines incubated with FCS-containing medium (Hanania et al, 1992; Bolsen et al, 1996).

In the case of topical ALA-application, the accumulation of higher levels of total porphyrins in tumours and psoriasis lesions appears to be the major effect responsible for the intralesional photosensitization. Our biochemical data show that the porphyrin metabolite patterns are not significantly changed upon ALA treatment.

Intravenous injection or oral administration of ALA was shown to optimize the homogeneous intralesional accumulation of porphyrins in PS, BCC and oral cavity SCC (Grant et al, 1993; Peng et al, 1995; Stringer et al, 1996). In mice or rats bearing colon or mammary carcinomas and in hamsters bearing an amelanotic melanoma, systemically administered ALA led to earlier (1 h) and higher tumour/skin ratios (6–30) of porphyrins than did topical ALA application (Peng et al, 1992; Hua et al, 1995; Sroka et al, 1996; Fritsch et al, 1997*a*). However, prolonged accumulation of porphyrins in the liver following intravenous injection of ALA was detected (Fritsch et al, 1997*a*) which may be responsible for reported side effects such as nausea, vomiting or transient increases in serum aspartate aminotransferase (Regula et al, 1994; Peng et al, 1995). Thus, topical application of ALA seems to be still the most promising technique in PDT of epithelial skin tumours due to lack of severe side effects (Fritsch et al, 1996*b*). PDT efficacy may be enhanced using ALA esters (Fritsch et al, 1998).

After topical application of ALA (for PDD or PDT), light avoidance for 2 days is recommended due to the prolonged porphyrin sensitization of normal skin, which lasts up to 48 h with decreasing intensity (data not shown). Psoriatic lesions may not offer

© Cancer Research Campaign 1999

optimum conditions for topical ALA-PDT and should be selected according to the fluorescence pattern under Wood's light.

### ABBREVIATIONS

ALA, δ-aminolaevulinic acid; BCC, basal cell carcinoma; DMSO, dimethylsulphoxide; EDTA, ethylenediaminetetraacetic acid; NS, normal skin; NS-A, normal skin adjacent to tumour or psoriasis lesion; NS-D, normal skin distant from tumour or psoriasis lesion; PDD, photodynamic diagnosis; PDT, photodynamic therapy; PS, psoriasis; SCC, squamous cell carcinoma

### ACKNOWLEDGEMENTS

CF has been supported by a grant of the Deutsche Forschungsgemeinschaft (Fr 1174/1–1).

### REFERENCES

Abels C, Heil P, Dellian M, Kuhnle GEH, Baumgartner R and Goetz AE (1994) In vivo kinetics and spectra of 5-aminolevulinic acid induced fluorescence in an amelanotic melanoma of the hamster. *Br J Cancer* **70**: 826–833

Bloomer JR, Brenner DA and Mahoney MJ (1997) Study of factors causing excess protoporphyrin accumulation in cultured skin fibroblasts from patients with protoporphyria. *J Clin Invest* **60**: 1354–1361

Boehncke WH, Sterry W and Kaufmann R (1994) Treatment of psoriasis by topical photodynamic therapy with polychromatic light. *Lancet* **343**: 801

Bolsen K, Lang K, Verwohlt B, Fritsch C and Goerz G (1996) In vitro induction of porphyrin biosynthesis in various human cells after incubation with δ-aminolevulinic acid. *Arch Dermatol Res* **288**: 320A

Calzavara-Pinton PG (1995) Repetitive photodynamic therapy with topical δ-aminolevulinic acid as an appropriate approach to the routine treatment of superficial non-melanoma skin tumours. *J Photochem Photobiol B*: *Biol* **29**: 53–57

Fritsch C, Becker-Wegerich PM, Schulte KW, Neuse W, Lehmann P, Ruzicka T and Goerz G (1996*a*) Treatment of a large superficial basal cell carcinoma of the breast with combination of photodynamic therapy and surgery controlled by photodynamic diagnosis. *Hautarzt* **47**: 438–442

Fritsch C, Verwohlt B, Bolsen K, Ruzicka T and Goerz G (1996*b*) Influence of topical photodynamic therapy with 5-aminolevulinic acid on the porphyrin metabolism. *Arch Dermatol Res* **288**: 517–521

Fritsch C, Abels C, Goetz G, Stahl W, Bolsen K, Ruzicka T, Goerz G and Sies H (1997*a*). Porphyrins preferentially accumulate in a melanoma following intravenous injection of 5-aminolevulinic acid. *Biol Chem* **378**: 51–57

Fritsch C, Neuse W, Ruzicka T and Goerz G (1997*b*) Photodynamische Diagnostik in der Dermatologie. In *Handbuch der Dermatologischen Phototherapie und Photodiagnostik*. Krutman J, Hönigsmann H (eds) pp. 331–356. Springer: Berlin

Fritsch C, Batz J, Bolsen K, Schulte KW, Zumdick M, Ruzicka T and Goerz G (1997*c*) Ex-vivo application of ALA induces high and specific porphyrin levels in human skin tumors. Possible basis for selective photodynamic therapy *Photochem Photobiol* **66**: 114–118

Fritsch C, Goerz G and Ruzicka T (1997*d*) Photodynamic therapy in dermatology. A review. *Arch Dermatol* **134**: 207–214

Fritsch C, Homey B, Stahl W, Lehmann P, Ruzicka T and Sies H (1998) Preferential relative porphyrin enrichment in solar keratoses upon topical application of δ-aminolevulinic acid methylester. *Photochem Photobiol* **68**: 218–221

Goerz G, Link-Mannhardt N, Bolsen K, Zumdick M, Fritsch C and Schürer NY (1995) Porphyrin concentrations in various human tissues. *Exp Dermatol* **4**: 218–228

Goetz AE Lumper W, Fritsch C, Müller W, Feyh J, Conzen P and Brendel W (1990) Changes in tumor and adjacent tissue perfusion after photodynamic therapy. *Eur Surg Res* **22**: (Suppl 1): 59

Goff B, Bachor R, Kollias N, Hasan T (1992) Effects of photodynamic therapy with topical application of 5-aminolevulinc acid on normal skin of hairless guinea pigs. *J Photochem Photobiol B* **15**: 139–251

Grant WE, Hopper C, Macrobert AJ, Speight PM and Bown SG (1993) Photodynamic therapy of oral cancer: photosensitisation with systemic aminolevulinic acid. *Lancet* **342**: 147–148

Hanania J and Malik Z (1992) The effect of EDTA and serum on endogenous porphyrin accumulation and photodynamic sensitization of human K562 leukemic cells. *Cancer Lett* **65**: 127–131

Hua Z, Gibson SL, Foster TM and Hilf R (1995) Effectiveness of δ-aminolevulinic acid-induced protoporphyrin as a photosensitizer for photodynamic therapy in vivo. *Cancer Res* **55**: 1723–1731

Iinuma S, Bachor R, Flotte T, Hasan T (1995) Biodistribution and phototoxicity of 5-aminolevulinic acid-induced PpIX in an orthotopic rat bladder tumor model. *J Urology* **153**: 802–806

Kennedy JC, Pottier RH and Pross DC (1990) Photodynamic therapy with endogenous protoporphyrin IX: basic principles and present clinical experience. *J Photochem Photobiol* **6**: 143–148

Kriegmair M, Baumgartner R, Knüchel R, Stepp H, Hofstädter F and Hofstetter A (1996) Detection of early bladder cancer by 5-aminolevulinic acid induced porphyrin fluorescence. *J Urol* **155**: 105–109

Lowry OH, Rosenbrough NJ, Farr AL and Randall RJ (1951) Protein measurement with the folin phenol reagent. *J Biol Chem* **193**: 265–275

Malik Z, Kostenich G, Roitman L, Ehrenberg B and Orenstein A (1995) Topical application of 5-aminolevulinic acid, DMSO and EDTA: protoporphyrin IX accumulation in skin and tumours of mice. *J Photochem Photobiol B*: *Biol* **28**: 213–218

Martin A, Tope WD, Grevelink JM, Starr JC, Fewkes JL, Flotte TJ, Deutsch TF and Anerson RR (1995) Lack of selectivity of protoporphyrin IX fluorescence for basal cell carcinoma after topical application of 5-aminolevulinic acid: implications for photodynamic therapy. *Arch Dematol Res* **287**: 665–674

Pass HI (1993) Photodynamic therapy in oncology: mechanisms and clinical use. *J Natl Cancer Inst* **85**: 443–453

Peng Q, Moan J, Warloe T, Nesland JM and Rimington C (1992) Distribution and photosensitizing efficiency of porphyrins induced by application of exogenous 5-aminolevulinic acid in mice bearing mammary carcinoma. *Int J Cancer* **52**: 433–443

Peng Q, Warloe T, Moan J, Heyerdahl H, Steen HB, Nesland JM and Giercksky GE (1995). Distribution of 5-aminolevulinic acid-induced porphyrins in noduloulcerative basal cell carcinoma. *Photochem Photobiol* **62**: 906–913

Regula J, Macrobert AJ, Gorchein A, Buonaccorsi GA, Thorpe SM, Spencer GM, Hatfield ARW and Bown SG (1994) Photosensitization and photodynamic therapy of esophageal, duodenal, and colorectal tumours using 5-aminolaevulinic acid induced protoporphyrin IX – a pilot study. *Gut* **36**: 67–75

Seubert A and Seubert S (1982) High-performance liquid chromatographic analysis of porphyrins and their isomers with radial compression columns. *Anal Biochem* **124**: 303–307

Sroka R, Beyer W, Gossner L, Sassy T, Stocker S and Baumgartner R (1996) Pharmacokinetics of 5-aminolevulinic-acid-induced porphyrins in tumour-bearing mice. *J Photochem Photobiol B*: *Biol* **34**: 13–19

Stringer MR, Collins P, Robinson DJ, Stables GI and Sheehan-Dare RA (1996) The accumulation of protoporphyrin IX in plaque psoriasis after topical application of 5-aminolevulinic acid indicates a potential for photodynamic therapy. *J Invest Dermatol* **107**: 76–81

Svanberg K, Anderson T, Killander D, Wang I, Stenram U, Andersson-Engels S, Berg R, Johansson J and Svanberg S (1994) Photodynamic therapy of non-melanoma malignant tumours of the skin using topical δ-amino levulinic acid sensitization and laser irradiation. *Br J Dermatol* **130**: 743–751

Szeimies RM, Sassay T and Landthaler M (1994) Penetration potency of topical applied delta aminolevulinic acid for photodynamic therapy of basal cell carcinoma. *Photochem Photobiol* **59**: 73–76

Veen van der N, Bruijn de HS, Berg RJW, Star WM (1996) Kinetics and localisation of PpIX fluorescence after topical and systemic ALA application, observed in skin and skin tumours of UVB-treated mice. *Br J Cancer* **73**: 925–930

Warloe T, Peng Q, Steen HB and Giercksky KE (1992) Localization of porphyrins in human basal cell carcinoma and normal skin tissue induced by topical application of 5-aminolevulinic acid. In *Photodynamic Therapy and Biomedical Lasers*. Spinelli P, Del Fante M, Marchesini R (eds) pp. 454–458. Elsevier Science Publishers B.V., Milan

Wolf P, Rieger E and Kerl H (1993) Topical photodynamic therapy with endogenous porphyrins after application of 5-aminolevulinic acid. *J Am Acad Dermatol* **28**: 17–21

© Cancer Research Campaign 1999
